# Critical Issues on the Surface Functionalization of Plasmonic Au-Ag/TiO_2_ Thin Films with Thiolated Oligonucleotide-Based Biorecognition Elements

**DOI:** 10.3390/bios14040159

**Published:** 2024-03-27

**Authors:** Diogo Costa, Patrícia Pereira-Silva, Paulo Sousa, Vânia Pinto, Joel Borges, Filipe Vaz, Graça Minas, Paula Sampaio

**Affiliations:** 1 Center of Molecular and Environmental Biology (CBMA), Department of Biology, University of Minho, 4710-057 Braga, Portugalid9456@uminho.pt (P.P.-S.); psampaio@bio.uminho.pt (P.S.); 2 Physics Center of Minho and Porto Universities (CF-UM-UP), Campus de Azurém, University of Minho, 4800-058 Guimarães, Portugal; fvaz@fisica.uminho.pt; 3 Center for Microelectromechanical Systems (CMEMS-UMinho), Campus de Azurém, University of Minho, 4800-058 Guimarães, Portugal; psousa@dei.uminho.pt (P.S.); vpinto@dei.uminho.pt (V.P.); gminas@dei.uminho.pt (G.M.); 4 LABBELS—Associate Laboratory, 4800-122 Braga, Portugal, and 4800-058 Guimarães, Portugal; 5 LaPMET—Laboratory of Physics for Materials and Emergent Technologies, University of Minho, 4710-057 Braga, Portugal; 6 Material Science Department, Transilvania University of Brasov, 29 Eroilor Blvd., 500036 Brasov, Romania

**Keywords:** reactive magnetron sputtered, thin films, Au-Ag NPs, localized surface plasmon resonance sensing, surface functionalization, synthetic thiolated oligonucleotides

## Abstract

This work reports on the surface functionalization of a nanomaterial supporting localized surface plasmon resonances (LSPRs) with (synthetic) thiolated oligonucleotide-based biorecognition elements, envisaging the development of selective LSPR-based DNA biosensors. The LSPR thin-film transducers are composed of noble metal nanoparticles (NPs) embedded in a TiO_2_ dielectric matrix, produced cost-effectively and sustainably by magnetron sputtering. The study focused on the immobilization kinetics of thiolated oligonucleotide probes as biorecognition elements, followed by the evaluation of hybridization events with the target probe. The interaction between the thiolated oligonucleotide probe and the transducer’s surface was assessed by monitoring the LSPR signal with successive additions of probe solution through a microfluidic device. The device was specifically designed and fabricated for this work and adapted to a high-resolution LSPR spectroscopy system with portable characteristics. Benefiting from the synergetic characteristics of Ag and Au in the form of bimetallic nanoparticles, the Au-Ag/TiO_2_ thin film proved to be more sensitive to thiolated oligonucleotide binding events. Despite the successful surface functionalization with the biorecognition element, the detection of complementary oligonucleotides revealed electrostatic repulsion and steric hindrance, which hindered hybridization with the target oligonucleotide. This study points to an effect that is still poorly described in the literature and affects the design of LSPR biosensors based on nanoplasmonic thin films.

## 1. Introduction

Localized surface plasmon resonance (LSPR) is a phenomenon observed when metallic nanoparticles (NPs), typically gold or silver, interact with light [1,2]. This interaction results in the collective oscillation of free electrons at the NP surface [3], leading to the enhanced absorption and scattering of light [4,5]. The resonance frequency in LSPR is tunable and depends on various factors including the size, shape, composition, and local environment surrounding the NPs [6,7]. These unique optical properties have found applications in sensing [8,9], spectroscopy [10,11,12], imaging [13,14], and photothermal therapy [15,16], among others.

In the past few years, LSPR-based transducers have been exploited for the development of biosensors [17,18,19], but although powerful, they lack inherent selectivity toward specific analytes [20,21]. Biorecognition elements such as antibodies, enzymes, and oligonucleotides are employed to impart selectivity to biosensors [22,23]. The use of thiolated (R-SH) molecules is the preferred method for bonding biorecognition elements to noble metal NP surfaces [24,25]. The interaction between the Au NP surface and sulfur atoms in thiol-containing molecules has gained significant breakthroughs in the field of nanobiotechnology [26] as it enables the precise and stable attachment of biorecognition elements [27]. This covalent linkage, known as the thiolate-gold bond, ensures robust attachment and preserves the activity of the biorecognition elements [28].

The conventional synthesis of NPs displaying LSPR involves the chemical reduction of metal salts, seed-mediated growth approaches [29,30], or physical methods such as laser ablation and sputtering [31,32,33]. However, thin films containing these NPs dispersed in a dielectric matrix offer versatile platforms for LSPR-based transducers [34]. Reactive direct current (DC) magnetron sputtering deposition, followed by thermal annealing treatment to induce the growth of NPs, provides a successful method to prepare nanocomposite surfaces sensitive to environmental changes [35].

In a previous study, the functionalization of Au/TiO_2_ thin films was reported, aiming at the production of sensitive LSPR-based optical transducers [36]. Streptavidin and biotin conjugated with horseradish peroxidase (HRP) were chosen as the model receptor–analyte to prove the efficiency of the immobilization method and to demonstrate the potential of the LSPR-based biosensor. However, it was found to have a relatively low LSPR response associated with the low sensing ability of the Au/TiO_2_ LSPR platform (bulk refractive index sensitivity, RIS = 33 nm/RIU). Thus, the developed sensing platform only presented an LSPR response in the presence of the ideal concentration of the analyte. Therefore, the sensitivity enhancement of these nanocomposite thin films has been a primary goal in subsequent work [34]. This implied tailoring the film’s structure and composition by changing different production conditions to enhance the LSPR signal, and hence the sensitivity. To achieve this, the deposition process was optimized, which includes incorporating bimetallic Au-Ag NPs and optimizing preparation conditions. Therefore, the sensitivity of the LSPR-based platform was greatly improved compared to the previous work mentioned [36].

The present work explores the functionalization of optimized nanoplasmonic Au-Ag/TiO_2_ thin-film transducers with thiolated oligonucleotide probes. A microfluidic device module was fabricated in polydimethylsiloxane (PDMS) to deliver the solution, allowing the study of the thin film’s surface/probe interaction by LSPR spectroscopy in real-time.

The use of oligonucleotides as biorecognition elements can provide a more direct approach to biosensing without using intermediaries such as biotin-streptavidin. A synthetic oligonucleotide, which is a sequence like the genome of the pathogen *Legionella pneumophila*, was selected as the recognition element, envisaging the development of sensitive and selective LSPR-based DNA biosensors. *L. pneumophila* is the Gram-negative bacterium responsible for Legionnaires’ disease, a severe respiratory ailment transmitted through contaminated water droplets [37,38,39]. The global increase in *L. pneumophila* epidemics emphasizes the need for advanced diagnostic methods [40,41], given the limitations of traditional methods such as culture-based testing and PCR [42,43,44].

## 2. Materials and Methods

### 2.1. Preparation and Characterization of Plasmonic Thin Films Containing Noble Metal NPs (Au, Ag) Dispersed in TiO_2_

#### 2.1.1. Thin-Film Deposition by DC Reactive Magnetron Sputtering

Custom-made reactive DC magnetron sputtering equipment was used to prepare the nanoplasmonic thin films composed of Au and Au-Ag NPs dispersed in a TiO_2_ dielectric matrix. The sputtering target was composed of titanium (99.99% purity) in a rectangular shape (200 × 100 mm^2^) with noble metal pellets placed in the target’s erosion track. To produce Au/TiO_2_ thin films, two pellets of Au that were 8 mm^2^ each were symmetrically placed in the titanium target. To obtain a thin film with an approximate 1:1 ratio of Au to Ag, a pellet of 8 mm^2^ of Au and a pellet of 4 mm^2^ of Ag were placed centered with the target erosion track. This area relation was achieved in previous optimizations of the films. The target was sputtered at a current density of 75 A/m^2^ in a plasma composed of Ar (3.8 × 10^−1^ Pa) and O_2_ (3 × 10^−2^ Pa) for 12 min to obtain a thin-film thickness of approximately 40 nm for both the Au/TiO_2_ and Au-Ag/TiO_2_ films (see Supplementary Materials for cross-sectional micrographs). The working pressure was 4.1 × 10^−1^ Pa, while the base pressure of the system was always below 6.0 × 10^−4^ Pa.

The thin films were deposited onto fused quartz (SiO_2_), quality JS1 purchased from Neyco Vacuum and Materials (92170 Vanves, France), with 25.0 × 9.0 mm^2^ for characterization, optical measurements, and immobilization experiments, and NaCl for the STEM analysis. The substrates were previously cleaned and activated with plasma using a Zepto plasma system (Diener Electronic, Ebhausen, Germany) with a 13.56 MHz generator at 50 W of power, first in an O_2_ atmosphere (60 Pa) for 5 min, followed by an Ar atmosphere (60 Pa) for 15 min.

#### 2.1.2. In-Vacuum Thermal Annealing Treatment to NPs Growth

The thin films were subjected to a post-deposition annealing treatment to induce the formation and growth of NPs throughout the TiO_2_ matrix and achieve LSPR responses. This thermal treatment was performed in a vacuum furnace with a base pressure of 8 × 10^−6^ Pa. The protocol consists of a heating ramp, steps of 5 °C/min until it reaches the desired temperature, an isothermal period of 5 h at 400 °C, and a last step of free cooling until it reaches room temperature.

#### 2.1.3. Chemical, Morphological, and Optical Characterization of Nanoplasmonic Thin Films

Rutherford backscattering spectrometry (RBS) was used to confirm the in-depth chemical composition of the thin films that were produced. The measurements were performed with a 2 MeV ^4^He^+^ beam, spotted in a 1 mm^2^ area, with angles of incidence of 0° (normal incidence) and 25°. Three detectors were placed in the equipment chamber: a standard at 140° and two pin-diode detectors located symmetrical to each other at 165° of scattering angle. The RBS data were analyzed with Ion Beam Analysis DataFurnace NDF v9.6i and according to algorithms developed by Barradas et al. [45,46,47].

Transmittance spectra of the thin films were measured before and after annealing in the range of 300 to 900 nm to confirm the presence of LSPR bands. These measurements were made on a UV-2450(PC) spectrophotometer from Shimadzu Corp (Kyoto, Japan).

Transmission electron microscopy (TEM) was used in the morphological analysis to obtain images of the NPs after thin-film annealing. This was performed under high-vacuum with an FEI-TITAN ETEM microscope from the INSA-Lyon Institute (Villeurbanne, France). The analysis was performed in high-angle annular dark-field scanning transmission electron microscopy (HAADF-STEM) mode. Additionally, the noble metals present on the bimetallic nanoplasmonic thin films were mapped with electron diffraction spectroscopy (STEM-EDX) using an X-MAX EDX detector from Oxford Instruments (Abingdon, UK) to demonstrate the presence of bimetallic Au-Ag nanoparticles in the thin film.

### 2.2. Fabrication of the Microfluidic Device and Connection to LSPR Spectroscopy System

Two different components were developed and fabricated for the immobilization experiments: the microfluidic channel, responsible for delivering the SH-oligonucleotide probe solution to the desired area using minimal quantities, and a custom optical measurement apparatus to couple with the LSPR spectroscopy system: a modular spectrophotometer and light emitting diode (LED) light source.

#### 2.2.1. Microfluidic Channel Development

**Figure 1:**
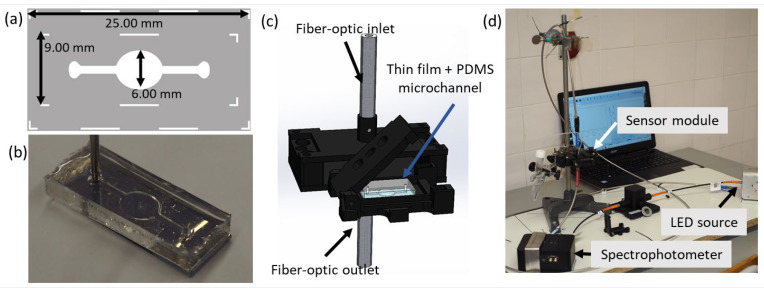
Fabricated components for the LSPR biosensor: (**a**) CAD for the microfluidic channel (this drawing was laser printed in a photomask and used in the UV exposure of SU-8); (**b**) microfluidic channel sealed against the thin film; (**c**) CAD of the sensor module; and (**d**) 3D printed sensor module with the light source and spectrophotometer assembled.

The microfluidic channel was fabricated in PDMS by replica molding using SU-8 photosensitive resin molds, fabricated through a cost-effective ultraviolet (UV) photolithography process without the need for cleanroom facilities and using conventional UV exposure equipment [48]. The microfluidic channels were designed using computer-aided design (CAD) software (Autodesk Fusion 360 v.2.0.18719) and printed in photomasks, which were used in the UV photolithographic process. The dimensions of the microfluidic channel are depicted in Figure 1a. The thickness of the fabricated microfluidic channel was approximately 100 µm.

The PDMS microfluidic channel was sealed on the thin film using O_2_ plasma treatment. Both the thin films and the microfabricated PDMS fluidic channel were exposed to O_2_ plasma (60 Pa) for 30 s at 15 W to promote the bonding of the microfluidic channel to the thin-film surface (Figure 1b), following an established protocol [49].

#### 2.2.2. Microfluidic Module Development and Alignment with LSPR Spectroscopy System

For the optical measurement system, a custom homemade holder was designed specifically to hold the PDMS microfluidic channel and ensure their alignment with the optical detection system using CAD software SolidWorks 2022 SP02. It was further 3D printed in an RBX01 3D printer from Cel Robox (Portishead, United Kingdom) (Figure 1c,d).

#### 2.2.3. Transmittance LSPR Spectroscopy System (Hardware and Software)

A white LED light source and a RES+ modular spectrophotometer, both purchased from SARSPEC, Lda. (Vila Nova de Gaia, Portugal), were connected by optical fibers to the sensing module, allowing real-time monitoring of the immobilization experiments with SH-oligonucleotide. The spectrophotometer was especially designed for acquiring high-resolution data in the wavelength range of 420 to 720 nm, where the LSPR response of Au and Au-Ag NPs in the TiO_2_ matrix occurs. The spectra were acquired with an integration time of 3 ms and an average of 200 scans. The resulting data were processed using NANOPTICS v.31 [50] software, which allows for quick processing of the data by fitting a 9th-order polynomial function to the spectral data, and follows the evolution of the LSPR band with the presence of DNA-based biorecognition elements.

### 2.3. Immobilization of Thiolated Oligonucleotide on Nanoplasmonic Thin Films

#### 2.3.1. Probe Selection

For the immobilization experiments, Tris-EDTA (TE) buffer was prepared with a pH of 8.0 for use as a washing solution and to prepare and store the aliquots. As a biorecognition element, a synthetic single-stranded oligonucleotide probe of the macrophage infectivity potentiator (*mip*) gene was selected (5′-ATAGCATTGGTGCCGATTTGGGGAAGAA). This sequence was chosen because it is only present in the *mip* gene of *Legionella pneumophila*, rendering the probe species-specific, and it has already been validated in the literature in the development of biosensors for the detection of this pathogenic bacteria [51,52]. This synthetic single-stranded oligonucleotide was purchased from Eurofins Genomics with a thiolated 3′ end (SH-oligonucleotide). A complementary oligonucleotide sequence (target-oligonucleotide) for the SH-oligonucleotide (5′ TTCTTCCCCAAATCGGCACCAATGCTAT) was also purchased from Eurofins Genomics, to be tested as the analyte.

#### 2.3.2. Optimization of Immobilization Conditions Using LSPR Sensing Response

Before the immobilization attempts with the biorecognition elements, the thin films were plasma-activated using Ar plasma treatment (Diener Electronic, Ebhausen, Germany) to erode the film’s surface, exposing the embedded NPs, as reported in previous studies [53].

Preliminary testing of SH-oligonucleotide immobilization on the surface of the nanoplasmonic thin film was conducted. For both Au and Au-Ag/TiO_2_ thin films annealed at 400 °C, the assay started with baseline measuring with TE buffer (pH 8.0) for 15 min, followed by the addition of 0.1, 0.5, and 1.0 nmol of SH-oligonucleotide each for 60 min, followed by washing the microfluidic channel with TE buffer.

The following experiment consisted of determining the probe’s saturation point on the surface of the NPs. For this, a baseline with TE buffer (pH 8.0) was measured for 35 min. Then, 0.2 nmol of SH-oligonucleotide was successively added through the microfluidic channel every 30 min until it reached a total of 1.0 nmol on the surface of the thin film. Finally, the system was washed with TE buffer (pH 8.0).

The effect of the incubation time was evaluated by the one-time addition of 0.5 nmol of SH-oligonucleotide to the thin film and monitoring the LSPR band for 90 min. The system was washed with TE buffer (pH 8.0) to end the surface reactions.

The detection of the target-oligonucleotide sequence was tested in the thin films functionalized with an SH-oligonucleotide probe by adding 0.1 nmol of the analyte through the microfluidic channel every 30 min after a baseline with TE buffer (pH 8.0) for 15 min. Afterward, the system was washed with TE buffer.

Each experiment was performed several times independently. The displayed data were consistent within the experiment.

#### 2.3.3. Hybridization Events Studies with Complementary Oligonucleotide Probes

For confirmation of the complementarity and hybridization of the SH-oligonucleotide probe to the target-oligonucleotide, a native polyacrylamide gel electrophoresis (PAGE) was performed. A gel with 20% polyacrylamide was prepared. As a DNA marker, the DNA ladder 1 kb Plus DNA Ladder from Thermofisher was used. Three wells of the electrophoresis were used: one for SH-oligonucleotide, another for the target-oligonucleotide sequence, and the last for a mix of the SH-oligonucleotide and target-oligonucleotide sequence. The coloration was performed by silver staining [54].

## 3. Results

### 3.1. Nanoplasmonic Thin Film Characterization

In this work, two thin-film systems were selected as LSPR-based sensing platforms, namely Au-Ag/TiO_2_ thin films with enhanced sensitivity, as demonstrated in previous work [34] as well as Au/TiO_2_ for comparison. For Au/TiO_2,_ RBS analysis showed a simulated thickness of about 40 nm and a Au concentration of 19 at.% in the TiO_2_ thin film. Regarding the Au-Ag/TiO_2_ thin film, it showed a total noble metal content of 17 at.%, with 9 at.% of Au, 8 at.% of Ag, and a similar thickness. SEM analysis corroborated the thickness simulated by the RBS technique, and the corresponding micrographs can be found in the Supplementary Materials.

**Figure 2:**
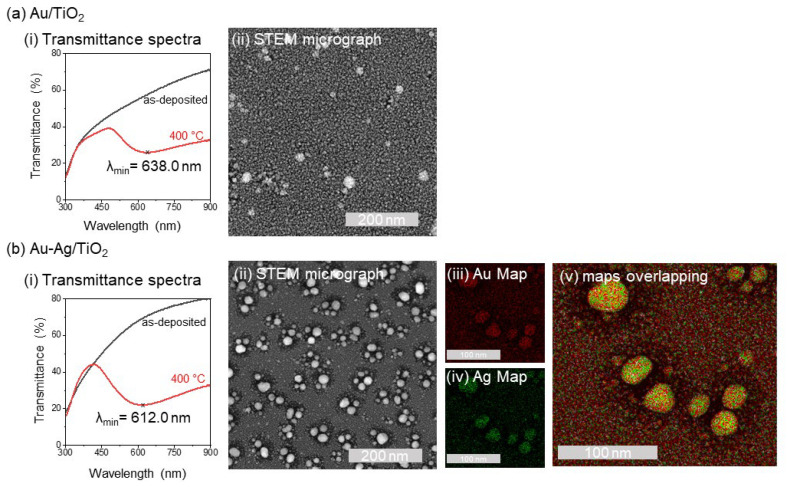
Optical and morphological analysis of (**a**) Au/TiO_2_: (i) transmittance spectrum (300 to 900 nm) for nanoplasmonic thin films before (black) and after annealing at 400 °C for 5 h (red), with the LSPR minimum marked; and (ii) HAADF-STEM micrographs for thin films annealed at 400 °C; and (**b**) Au-Ag/TiO_2_: (i) transmittance spectrum (300 to 900 nm) for nanoplasmonic thin films before (black) and after annealing at 400 °C for 5 h (red), with the LSPR minimum marked; (ii) HAADF-STEM micrographs for thin films annealed at 400 °C; (iii) Au and (iv) Ag STEM-EDX mapping, and (v) overlapping of Au and Ag maps over the original micrograph.

Confirming the presence of the LPSR band is crucial to the functionality of the thin-film transducer. Transmittance spectroscopy was used to evaluate the optical response of the produced thin films at the visible wavelength range. As expected, before the thermal treatment, the as-deposited thin films did not display observable LSPR bands in either the Au/TiO_2_ (Figure 2a) or Au-Ag/TiO_2_ (Figure 2b) compositions as the noble metal nanoparticles had not yet reached sizes above the quantum limit (i.e., above 10 nm) [55].

After in-vacuum annealing at 400 °C, both the Au/TiO_2_ and Au-Ag/TiO_2_ spectra evolved to display an LSPR band. The formation of nanoparticles can. be attributed to the (i) diffusion of noble metal atoms into nucleation sites, (ii) nucleus growth, and (iii) coalescence of adjacent nanoparticles (NPs). Noble metal NPs are responsible for supporting localized surface plasmons that can be excited by transmitted light [55] and are known to be sensitive to changes in the dielectric environment surrounding them, establishing the basis of LSPR-based sensors.

The results clearly showed that the LSPR band properties changed due to the introduction of Ag in the Au-TiO_2_ thin films annealed at 400 °C, leading to a blueshift in λ_min_ from 638.0 to 612.0 nm. This behavior resulted from the combination of Ag with Au in bimetallic nanoparticles, where the LSPR has an extinction maximum (transmittance minimum) in the range between the monometallic counterparts. Furthermore, it can also be demonstrated by Mie’s theory that Ag NPs reveal higher scattering and absorption efficiencies than Au, as also experimentally observed in previous studies [34]. Furthermore, it is also known that as the scattering efficiency increases, so does the responsiveness to the changes in the refractive index surrounding the NPs, so it is expected that synergetic characteristics of Ag and Au will enhance the LSPR sensitivity.

HAADF-STEM images also corroborate the optical measurement analysis. Figure 2(aii,bii) shows the overall difference in NP distribution within the thin film, with the bimetallic Au-Ag/TiO_2_ thin film exhibiting a higher number of NPs embedded in the TiO_2_ dielectric matrix. Furthermore, the STEM-EDX of Au (Figure 2(biii)) and Ag (Figure 2(biv)) allowed us to map the distribution of each element throughout the Au-Ag/TiO_2_ thin film, showing an overlap of Au and Ag (Figure 2(bv)), indicating the formation of bimetallic NPs.

### 3.2. Thin Film’s Surface Functionalization with SH-Oligonucleotide

#### 3.2.1. LSPR Sensing Response of Au/TiO_2_ and Au-Ag/TiO_2_ Thin Films during Probe Immobilization

In the biosensor field, the immobilization of thiolated biorecognition elements on the surfaces of Au NPs is a key research area. One of the main challenges involved in the progress of optical biosensors is the efficient capture of biorecognition elements and their transformation into detectable optical signals. For real-time monitoring of the LSPR signal, the optical system (HR-LSPR spectroscopy system) is described in Section 2.2 (Figure 1). The first step to initiate functionalization of the Au/TiO_2_ and Au-Ag/TiO_2_ thin films is to partially expose the embedded NPs using Ar plasma. The authors already demonstrated the process of partially exposing the NPs in a previous work [53], and it is expected to enhance the available bonding sites for SH-oligonucleotide probes by removing the hydrocarbon layers and eroding the most superficial layers of the TiO_2_ matrix. Next, microfabricated PDMS fluidic channels were bonded to the thin films using O_2_ plasma. For real-time monitoring of the LSPR band during the functionalization assays, the nanoplasmonic thin film, integrated with the PDMS microfluidic channel, was positioned in the sample holder.

**Figure 3:**
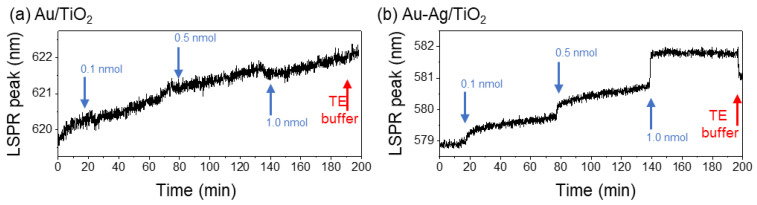
Evaluation of the nanoplasmonic thin films’ sensitivity to 0.1, 0.5, and 1.0 nmol of the SH-oligonucleotide probe (arrows): (**a**) Au/TiO_2_ and (**b**) Au-Ag/TiO_2_.

Before the initial analysis in both Au/TiO_2_ and Au-Ag/TiO_2_ nanoplasmonic systems, the optical system readout was stabilized, performing a continuous measurement of the LSPR signal for Au-Ag/TiO_2_ thin films without changing the surrounding medium of the NPs for 120 min (using the buffer solution), with no significant changes. Preliminary testing of both nanoplasmonic thin films involved testing the adsorption of three different amounts of SH-oligonucleotide (0.1, 0.5, and 1.0 nmol) to evaluate the thin films’ sensitivity in response to surface functionalization. Between each SH-oligonucleotide concentration, the probe was allowed to stabilize for 1 h. The results for the Au/TiO_2_ and Au-Ag/TiO_2_ thin films are presented in Figure 3.

The results obtained with the Au-TiO_2_ thin films annealed at 400 °C indicate that discerning shifts in the LSPR peak position due to SH-oligonucleotide addition are challenging due to the high background noise (Figure 3a). In contrast, Au-Ag/TiO_2_ thin films exhibited a much lower baseline noise than Au/TiO_2,_ and it was possible to observe the LSPR peak shift due to thiolated probe addition (Figure 3b). After each addition of the probe, these visible redshifts of the LSPR band demonstrated an increased local refractive index around the NPs. Moreover, it was possible to observe that after the addition of 1.0 nmol of the SH-oligonucleotide probe (1.6 nmol in total), the slope of the signal was near zero, suggesting a saturation effect. Based on these results, the Au/TiO_2_ thin film was excluded from further optimizations. Regarding the Au-Ag/TiO_2_ thin films, they saturated the SH-oligonucleotide bonds on the NPs at probe amounts between 0.6 and 1.6 nmol.

The sensing behavior agreed with the values estimated for the bulk refractive index sensitivity (RIS) determined for these two systems [56,57], where an almost two-fold increase from 80 ± 4 to 147.7 ± 0.9 nm/RIU was found when Ag was added to Au to form bimetallic nanoparticles. Therefore, as expected, a highly sensitive LSPR-based thin film is required to monitor such functionalization events.

#### 3.2.2. Optimizing SH-Oligonucleotide Immobilization as a Biorecognition Element

The next set of experiments was only conducted on the Au-Ag/TiO_2_ thin film annealed at 400 °C. First, the number of SH-oligonucleotide probes on the thin-film surface was increased by successively adding 0.2 nmol of probe solution, each for 30 min.

**Figure 4:**
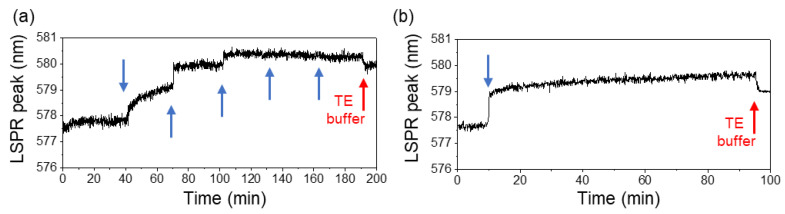
Surface functionalization experiments of the SH-oligonucleotide probe (arrows) on Au-Ag/TiO_2_ thin films annealed at 400 °C: (**a**) successive addition of 0.2 nmol of SH-oligonucleotide, each 30 min, to the microfluidic channel; and (**b**) addition optimized 0.5 nmol of the thiolated probe and incubation for 90 min.

The system was stabilized with TE buffer for 35 min before any addition of the SH-oligonucleotide. Figure 4a displays the evolution of the LSPR signal with the sequential addition of 0.2 nmol of SH-oligonucleotide probe through the microfluidic channel.

After injecting 0.2 nmol of the SH-oligonucleotide probe, the LSPR band notably redshifted, responding to the refractive index alteration surrounding the Au-Ag NPs. The system was left to stabilize for 30 min. During that period, it was clear that the signal was still shifting, suggesting a continuous interaction of the thiolated probe with the thin film’s surface (Figure 4a). The addition of another 0.2 nmol of SH-oligonucleotide (total of 0.4 nmol) led to observable LSPR signal responses. During the stabilization period, the LSPR band remained constant, suggesting that 0.4 nmol could be enough to saturate the transducer with the probe. The following addition, totaling 0.6 nmol of SH-oligonucleotide, slightly shifted the position of the LSPR band. Moreover, the additional SH-oligonucleotide did not introduce changes in the positioning of the LSPR band, indicating a potential saturation between 0.4 and 0.6 nmol. The subsequent washing of the system with TE buffer resulted in a minimal LSPR band change, a clear indication that the probes were firmly adhered to the thin-film surface. These would be washed out otherwise, and the LSPR signal would be shifted to the starting point. After TE buffer washing, the final LSPR signal was similar to that after introducing 0.4 nmol of SH-oligonucleotide, indicating that this might be the maximum concentration of probe that can be attached to the Au-Ag/TiO_2_ thin film.

Considering the saturation amount for SH-oligonucleotide immobilization onto the Au-Ag NPs at around 0.4 nmol, the reaction time was then assessed. As such, after stabilizing the thin-film LSPR signal with TE buffer for 10 min, 0.5 nmol of the probe was added to the Au-Ag/TiO_2_ thin film surface through the microfluidic channel. The SH-oligonucleotide solution was allowed to interact with the thin film surface for 90 min while monitoring the LSPR band (Figure 4b). The thin film’s surface was subsequently washed with TE buffer to remove unbonded probes. In the first 10 min of interaction between the thiolated probe and the thin film surface, the LSPR signal shifted from 577.7 to 579.3 nm. For the remaining time (80 min), the LSPR signal only shifted 0.3 nm, stabilizing at approximately 579.6 nm of wavelength. Washing the system with TE buffer promoted a slight blueshift in the LSPR signal, similar to the previous experiment. Furthermore, the signal remained consistently high (at approximately 579.1 nm), indicating the bonding of the SH-oligonucleotide to the Au-Ag NPs in the thin film.

These studies clearly showed a strong and fairly quick interaction between the SH-oligonucleotide and the film’s surface, evidenced by the persistent alteration of the LSPR band’s position in response to changes in the local refractive index near the surface of the NPs.

#### 3.2.3. Detection of Complementary DNA Sequence

**Figure 5:**
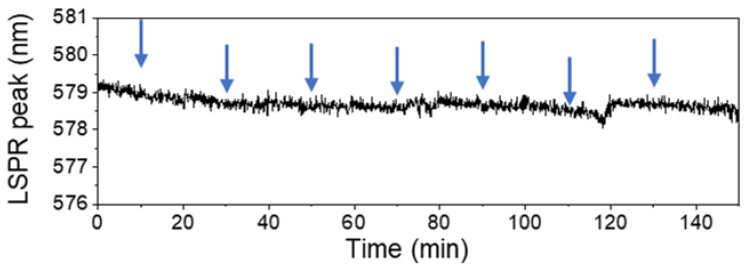
Detection of target-oligonucleotide sequence in the SH-oligonucleotide functionalized Au-Ag/TiO_2_ thin films (the arrow indicates the target-oligonucleotide sequence addition time).

The next step of the study was to detect the binding of the target-oligonucleotide, complementary to the SH-oligonucleotide probe attached to the Au-Ag NPs. As previously described, following the initial stabilization of the LSPR signal with TE buffer (pH 8.0), 0.1 nmol of the target-oligonucleotide was introduced through the PDMS microfluidic channel and allowed to hybridize for 20 min (Figure 5).

A total of 0.7 nmol of target-oligonucleotide was added. Finally, the system was washed with TE buffer. During the experiment, continuous monitoring of the LSPR band was performed using the described custom optical system. However, the detection of the attachment of the target-oligonucleotide to the SH-oligonucleotide probe through a shift in the LSPR signal was unsuccessful. No significant alterations in the LSPR band position were observed during the experiment including both the addition of the target-oligonucleotide and the subsequent washing step with TE buffer.

The lack of LSPR response might indicate several possibilities: the complementary target-oligonucleotide may not have successfully hybridized with the immobilized SH-oligonucleotide, the potential degradation of one or both the oligonucleotides, or the saturation of the LSPR signal with the excessive immobilized thiolated probe.

#### 3.2.4. Hypothesizing the Performance of LSPR Biosensor Observed during Detection of Target-Oligonucleotide

**Figure 6:**
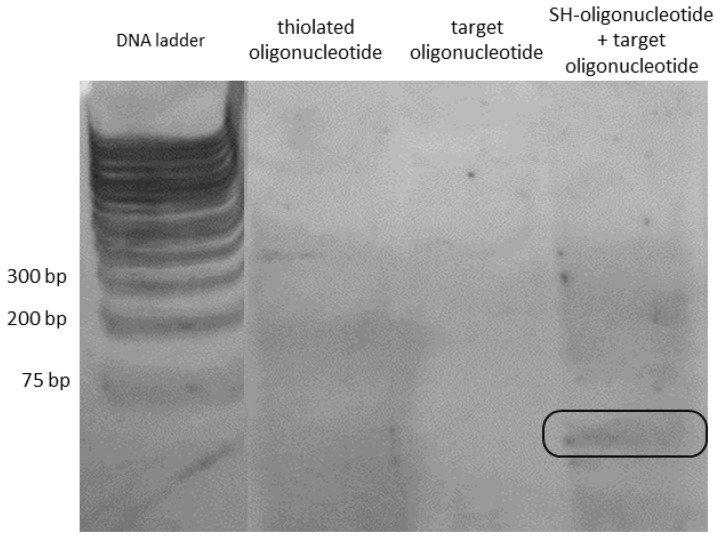
Native-PAGE for the SH-oligonucleotide and target-oligonucleotide probes.

To discern whether the SH-oligonucleotide probes were structurally compromised or incapable of hybridization, a native PAGE was performed and stained using a sensitive silver staining protocol (Figure 6).

The gel electrophoresis setup consisted of a 1 kb DNA ladder ranging from 75 to 1000 bp that was used to establish the running front and the probe’s positions. Subsequently, the SH-oligonucleotide and the target-oligonucleotide, each with 28 bases, were loaded separately into the subsequent wells. Finally, the SH-oligonucleotide and the complementary probe were mixed in an Eppendorf tube and allowed to hybridize for 30 min before being loaded into the last well of the polyacrylamide gel. The gel was run at a lower power of 200 mV and 35 mA for 60 min to accommodate the small molecular weight of both the SH-oligonucleotide probe and target-oligonucleotide in comparison with the DNA ladder. The results showed no evident bands in the wells concerning the single-strand probes and both the thiolated and complementary oligonucleotides after staining. A band of approximately 36 bp was measured for the hybridized oligonucleotides after silver staining (Figure 6), while the expected size was 28 bp for these probes. Additionally, due to the 3′ end modifications in both single-stranded sequences (the thiol group and biotin, respectively), the diffusion through the electrophoresis gel could have been delayed.

It is known that double-stranded DNA (dsDNA) is more effectively stained than single-stranded DNA after electrophoresis [58]. This may explain the lack of visualization of the separated probe in the gel compared to the hybridized probes. Consequently, this result allowed to dismiss the absence of hybridization and structural compromises of the probes as reasons for the inability of the nanoplasmonic thin film to detect the binding of the target-oligonucleotide. This outcome suggests that the inability to detect the binding might come from the functionalized surface of the Au-Ag/TiO_2_ thin film due to two major reasons.

On the one hand, a possible oversaturation of the film’s surface with SH-oligonucleotide probes might hinder hybridization events. On the other hand, the sensitivity of the developed LSPR-based biosensor (i.e., the transducer functionalized with the probes) might still be too low to monitor hybridization with complementary DNA. Regarding the latter (possible) effect, and according to Figure 4a, the Au-Ag NPs were able to discern the differences between the refractive index of the TE buffer (η = 1.354) and the SH-oligonucleotide added during the immobilization experiments. The estimated difference in refractive index between ssDNA (η = 1.456) and dsDNA (η = 1.530) [59,60] was similar to the difference between TE buffer refractive index and SH-oligonucleotide. As such, the nanoplasmonic thin film should be able to detect the change in refractive index due to the hybridization of complementary ssDNA to dsDNA.

This means that the lack of probe hybridization on the surface of the thin film must be the major issue to be considered. There is a known correlation between the density of ssDNA molecules adhered to the surface and its ability to form dsDNA molecules when the complementary DNA strand is present. Indeed, increasing the density of ssDNA molecules adhered to a surface can lead to the electrostatic repulsion of free DNA by the immobilized DNA strand and steric hindrance effect [61], making it difficult to form dsDNA molecules. Therefore, by saturating the thin-film transducer with SH-oligonucleotide probes, steric accessibility, electrostatic repulsion, or orientational constraints compromise the hybridization with the complementary oligonucleotide. Consequently, no LSPR band shift was observed.

## 4. Conclusions

In conclusion, the successful immobilization of SH-oligonucleotides (biorecognition elements) on the surface of Au-Ag/TiO_2_ thin films was followed by measuring the LSPR band shift over time. Saturation of the LSPR signal was found at approximately 0.4 nmol of the SH-oligonucleotide probe. Nevertheless, the Au-Ag/TiO_2_ nanoplasmonic system was unable to sense the hybridization of the biorecognition elements with the complementary target-oligonucleotide by LSPR sensing.

Gel electrophoresis suggested that the lack of an LSPR signal was not due to the lack of hybridization capability or compromised structural integrity of the probes. Knowing that the produced thin films can distinguish between the refractive indices of ssDNA vs. dsDNA, the issue could be the overpopulation of thiolated probes at the film’s surface. This could create difficulties for the hybridization of the DNA sequences, thus compromising the LSPR detection.

Subsequent studies (e.g., by using fluorescence-labeled complementary oligonucleotides) need to be performed to evaluate the SH-oligonucleotide occupation of the film’s surface to find an equilibrium between the amount of immobilized biorecognition elements and the ability to monitor hybridization events using LSPR biosensors based on Au-Ag/TiO_2_ plasmonic thin films.

## Data Availability

The data presented in this study are available on request from the corresponding author.

## Supplementary Materials

The following supporting information can be downloaded at: https://www.mdpi.com/article/10.3390/bios14040159/s1, Figure S1—SEM micrographs at different magnifications and thickness measurements for thin films annealed at 400 °C.
