# Supplementary material for: Critical Issues on the Surface Functionalization of Plasmonic Au-Ag/TiO2 Thin Films with Thiolated Oligonucleotide-Based Biorecognition Elements
Source: Biosensors (Basel). 2024 Mar 27;14(4):159. doi: 10.3390/bios14040159 (PMC11048063; doi:10.3390/bios14040159)
Supplement: Supplementary file 1 [file biosensors-14-00159-s001.zip › biosensors-2894406-supplementary.pdf]

Critical issues on the surface functionalization of plasmonic Au-Ag/TiO2 thin films with thiolated oligonucleotide-based biorecognition elements

Diogo Costa[1,2,3], Patrícia Pereira-Silva[1,2], Paulo Sousa[3,4], Vânia Pinto[3,4], Joel Borges[2,5,*], Filipe Vaz[2,5,6], Graça Minas[3,4], and Paula Sampaio[1]

SUPPLEMENTARY MATERIAL

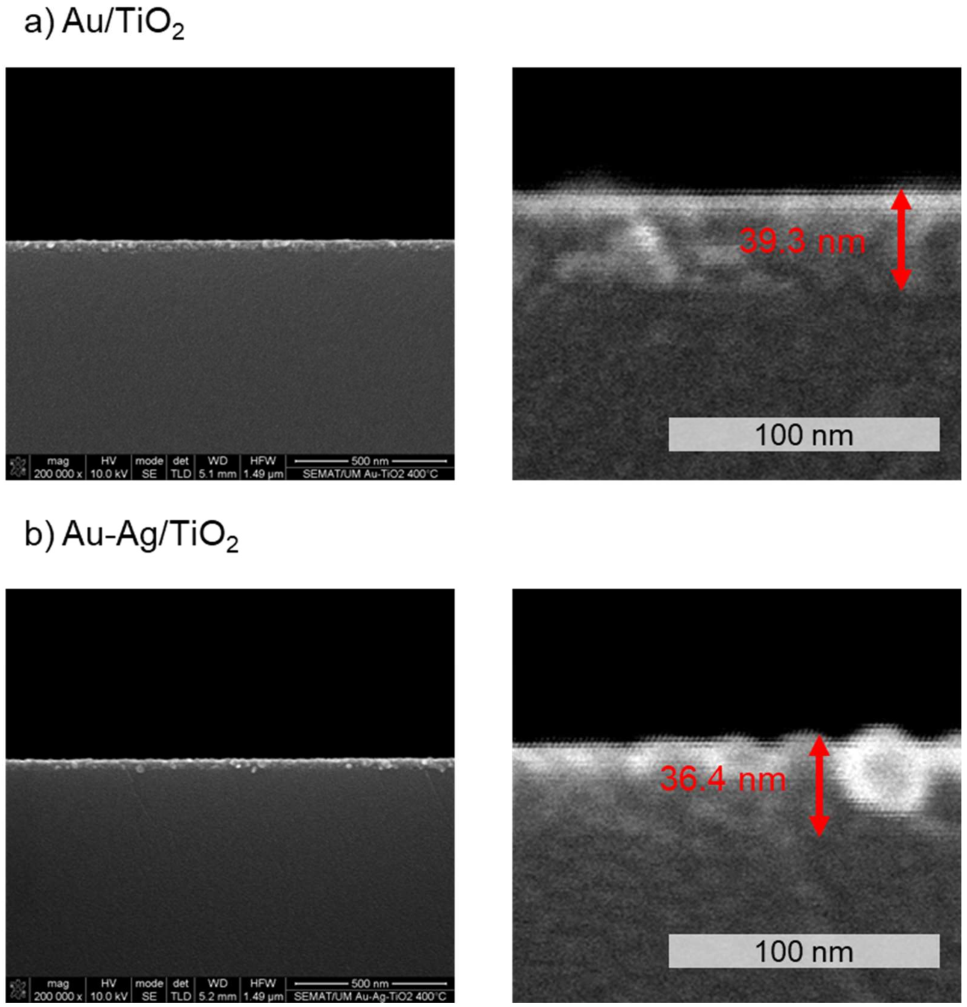

Figure S1 - SEM micrographs at different magnifications and thickness measurements for thin films annealed at 400 °C: a) Au/TiO_2 and b) Au-Ag/TiO_2. The images were obtained using a NanoSEM - FEI Nova 200 (FEG/SEM) scanning electron microscope at 10.0 kV.
